# A Metagenome-Based Methodology to Track Genomically Recoded Strains and Assess Their Effects on Indigenous Microbes

**DOI:** 10.1021/acs.est.5c15663

**Published:** 2026-07-20

**Authors:** Ana Durán-Viseras, Gyuhyon Cha, Janet K. Hatt, Blake G. Lindner, Efterpi M. Benvenuto, Yiran Zhang, Aditya M. Kunjapur, Konstantinos T. Konstantinidis

**Affiliations:** † School of Civil and Environmental Engineering, 1372 Georgia Institute of Technology, Atlanta, Georgia 30332, United States; ‡ Chemical and Biomolecular Engineering, University of Delaware, Newark, Delaware 19716, United States

**Keywords:** recoded microorganisms, artificial amino acids, synthetic biology, environmental microbiology, horizontal gene transfer, time-series metagenomes

## Abstract

Assessing the effects of the release of biologically contained microorganisms into the environment represents a challenging task as it requires both the tracking of escape events as well as the changes that result in the indigenous microbes, which cannot be effectively determined based on conventional culture-based methodologies. Toward closing this gap, we set up closed, laboratory mesocosms with water from a nearby recreational-use freshwater reservoir that were subsequently spiked with the *Escherichia coli* strain DEP to simulate an accidental spill of a synthetic organism into the environment. Strain DEP is a chloramphenicol-resistant synthetic auxotroph harboring three redesigned genes encoding nonstandard amino acid (nsAA)-dependent gene products for l-4,4′-biphenylalanine (BipA) dependence. Shotgun metagenome sequencing of the mesocosms revealed a sharp decline in the relative abundance of strain DEP over time, with minimal impact on the indigenous freshwater microbial communities as evidenced by the recovery of these communities to the preperturbation state after 2 days of incubation. Further, there were no observations of transfer of the nsAA-dependent genes to the indigenous populations at the limit of detection of our metagenome sequencing effort or based on culturing on BipA-supplemented media. Collectively, our results show that this particular strain DEP may not pose a serious environmental threat if accidentally released into the environment due to low competitiveness against the indigenous freshwater microbes and the lack of escape mutants. Notably, this work establishes a holistic approach to assess biocontainment efficacy that should be applicable to additional genetically modified organisms.

## Graphical abstract

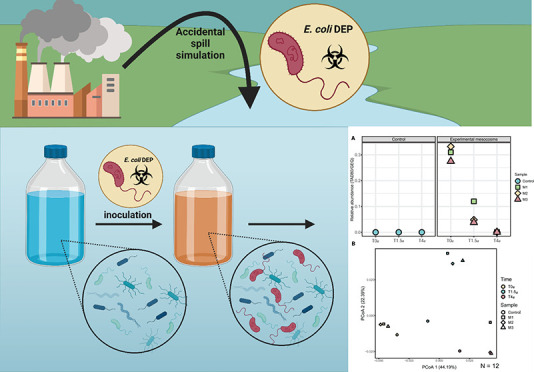

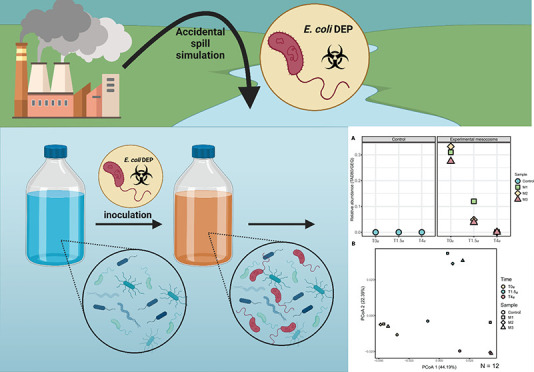

## Introduction

The field of synthetic biology (SynBio) is rapidly producing bioengineered microorganisms toward promising applications in bioremediation, biofuel production, and other biotechnological and medical applications. However, these advancements raise new concerns regarding potential threats posed by SynBio organisms to human health and environmental safety. These concerns emerge mostly from the (possible) persistence of these microorganisms in the environment, the transmission of their genetic material to indigenous organisms via horizontal gene transfer (HGT), or unpredictable interactions and further impacts on biodiversity and ecosystems upon their release.^1–3^ To help mitigate these risks, work in SynBio often includes efforts to genetically engineer microorganisms with safety switches, aimed to prevent escape and/or minimize their impact on the environments they are deployed to or accidentally released in. For example, some SynBio organisms have been designed with intrinsic biological containment mechanisms, such as genetic circuits that self-destruct in the absence of required inputs (“kill switches”) or metabolic dependency on a specific rare or synthetic nutrient (“auxotrophy” or “synthetic auxotrophy”), that should result in an inability to survive in the environment in the case of an accidental or deliberate release event.^4^ Understanding the time scale of persistence of biocontained organisms, their interactions with indigenous microbiota in natural ecosystems, and identifying any unintended consequences of their release is needed in order to assess any potential threats to human health and the environment.^3,5^ Addressing these gaps in knowledge will support the safe and responsible use of SynBio microorganisms in future applications.

The discipline of synthetic biology has generated a wide variety of genetically engineered organisms, ranging from those incorporating catabolic pathways from other organisms to those engineered with expanded genetic codes. In this study, we focus on a representative of the latter: the *E. coli* DEP strain, a synthetic auxotroph that is engineered to rely on the nonstandard amino acid (nsAA) l-4,4’′-biphenylalanine (BipA) for its growth. It harbors three essential genes that encode proteins computationally redesigned to rely on BipA for proper folding and function. In their native genomic loci are two synthetic auxotrophic markers: adenylate kinase (*adk.d6*) and tyrosyl-tRNA synthetase (*tyrS.d8*). Encoded within a plasmid (ori: p15a; *Cm* ^R^) is the third synthetic auxotrophic marker, a BipA-dependent aminoacyl-tRNA synthetase for aminoacylation of BipA (*bipARS.d6*), that serves as an essential protein only in strains that contain other markers reliant upon BipA such as strain DEP.^6^ That is, the activity of *bipARS.d6* is necessary for the production of functional Adk.d6 and TyrS.d8 proteins that are essential for the growth of DEP. Therefore, these synthetic markers could be classified into three categories: (1) on the genome and detrimental—*adk.d6* and *tyrS.d8*; (2) on the plasmid and neutral—*bipARS*/tRNA; and (3) on the plasmid and conditionally beneficial—chloramphenicol acetyltransferase (*Cm* ^R^).

Genetically modified organisms can escape defined media and growth limitations through mutagenic drift, environmental supplementation, or HGT.^7,8^ The frequency of such escape events is quantified as “escape frequency” representing the proportion of organisms that evade containment measures. “Escape frequencies”, based on culture assays, have been reported to range from 10^–6^ to undetectable levels below 10^–12^, depending on the robustness of the biocontainment strategies employed.^7^ Along this line, “synthetic auxotrophy” for non-natural compounds has proven to be an effective and robust biocontainment strategy against all three escape mechanisms.^8,9^ However, detecting and tracking SynBio microorganisms upon their release into the environment based on culture assays represents a challenging task; for instance, microorganisms often lose cultivability (but remain viable) upon changing growth conditions and environments.^10^ Metagenomics can offer a solution to this challenge by providing highly sensitive identification of target genomes *in situ*, including engineered strains, and assessing their associated ecological impact on the natural microbial communities upon release. Metagenomics could also possibly help in detecting the transfer of synthetic genes to the indigenous microbial populations (e.g., HGT).^11,12^

In this study, we followed a time course of the abundance of strain *Escherichia coli* DEP, a biocontained synthetic auxotroph derived from the genomically recoded *E. coli* strain C321.ΔA, inoculated into freshwater mesocosms with water from the Chattahoochee River (3 biological replicates), using both culture-based methods and shotgun metagenomics. Further, we assessed the effects of the spike-in strain on the indigenous microbial community, the strain’s time scale of persistence, and the potential for HGT of the genes carried by the biocontained strain to indigenous microbes in the freshwater inoculum. Our results expand the understanding of the interactions of this type of biocontained microorganism in the natural environment, suggesting minimal environmental risk for *Escherichia coli* DEP as a chassis for synthetic biology in the event of an accidental or deliberate release.

## Materials and Methods

### Genetically Modified *Escherichia coli* Strain “DEP”

The *Escherichia coli* genome used for the spike-in mesocosm experiment is a modified version of the genomically recoded strain C321.ΔA,^6^ designated as DEP. DEP was not modified in this study, but for clarity we briefly describe how it was constructed in a prior study. BipA-dependent aminoacyl-tRNA synthetase (BipARS.d7) was introduced into strain C321.ΔA, subsequently the endogenous *tdk* gene from C321.ΔA was deleted, the endogenous *adk* and *tyrS* genes were replaced with their codon-shuffled variants (*adk*(recode)-*tdk* and *tyrS*(recode)-*tdk*) transcriptionally fused to *tdk*, and the fusion cassettes were replaced with the *adk.d6* and *tyrS.d8* variants, as described previously.^8^

### Sampling and River Mesocosm Experiments

Surface water from the Chattahoochee River (1–2 m depth) was collected in sterile Nalgene 60 L carboys. The river water was subsequently filtered through 2.0 μm filters to remove large suspended solids. The resulting water after filtration was clear, and no sedimentation was observed. We did not perform physicochemical characterization of the water as part of this study; however, the bacterial community of the control resembled that of the surface river water based on our previously published metagenomes from the same area^13,14^ and we refer the reader to our previous publications for the water quality data. Our previous work has also shown that prefiltering water through a 2.0 μm filter does not substantially change the bacterial community in samples from this riverine ecosystem.^13,14^ For the mesocosm setup, sterile 20-L glass jugs were filled with 12 L of filtered river water and inoculated with 2.29E + 09 CFU, which resulted in an approximate concentration of 1.91E + 05 CFU/mL, of the *E. coli* DEP strain (n = 3, triplicate mesocosms: M1, M2, and M3). The fourth jug served as a control without DEP inoculation (C). Mesocosms were incubated in the dark at room temperature throughout the duration of the experiment and swirled every 12 h to create aerated conditions. Sampling occurred at 0, 0.5, 1, 1.5, 2.5, 3, 4, and 5 days by sacrificing 1 L of river water from each mesocosm and control. Each sample of water was filtered through a 0.22 μm filter, and the filter was stored at −80 °C until DNA extraction.

### Culturing, DNA Purification, and Shotgun Sequencing

For quantification of viable DEP cells, 50 μL of each sample was diluted 10-fold up to 1E + 06 and then plated in duplicate on permissive LB media supplemented with 25.5 μg/mL chloramphenicol, 0.2% l-arabinose, 20 mM Tris-HCl (pH= 7.5), 100 μM BipA, 0.005% SDS, and 1.5% agar. Plates were incubated in the dark at 35 °C for 2 days, and *Escherichia coli* CFUs were enumerated afterward (Table S1). For mesocosm inoculation, DEP cells were grown to desirable densities in the same LB medium described above but without agar, washed in PBS buffer to remove media, and subsequently added to the mesocosm. DNA from the three mesocosms (M1, M2, and M3) and the control (C) at time 0, 1.5, and 4 days after inoculation was extracted from filters using the DNeasy Powersoil Pro kit (Qiagen), followed by 2 × 150 bp paired-end shotgun sequencing on the Illumina NovaSeq 6000 platform at the Molecular Evolution Core of the Georgia Institute of Technology. Genomic DNA from bacterial isolates was sequenced using an in-house Oxford Nanopore MinION instrument. Briefly, libraries were prepared using the Rapid Barcoding Kit 24 (SQK-RBK114-24) following the manufacturer’s instructions, allowing multiplexed sequencing of multiple isolates on a single flow cell. Sequencing was performed on an R10.4.1 flow cell (FLO-MIN114).

### Sequencing Data Analysis

Raw short-read, Illumina data were quality-checked and trimmed using Multitrim (https://github.com/KGerhardt/multitrim). Trimmed reads were assembled independently *de novo* with SPAdes 3.15.4 (“-meta”)^15^ and IDBA-UD 1.1.3,^16^ and the generated assemblies were quality-checked using metaQUAST^17^ (metagenome statistics are available in Table S2). Contigs shorter than 5 Kbp from both assemblies were removed prior to population genome binning, which was performed independently for each assembly by MaxBin 2.2.7 and MetaBAT 2.12.1 with default parameters.^18,19^ Additionally, in a parallel workflow, trimmed reads were normalized by the BBNorm tool of the BBTools package,^20^ and subsequently assembled and binned as described above. All resulting Metagenome-Assembled Genomes (MAGs) from the same sample (both regular and depth-normalized short-read assemblies) and the different assembly and binning runs were dereplicated using dRep 3.4.0 ^21^ with a 95% ANI threshold. MAG contamination and completeness were assessed with CheckM 1.1.2.^22^ This process resulted in 19 MAGs representing the species-level diversity recovered in the samples (MAG statistics are available in Table S3). Base calling and demultiplexing of Nanopore isolate sequences were carried out using Dorado (v1.4.0) with the high-accuracy (HAC) model (dna_r10.4.1_e8.2_400bps_hac@v5.2.0). Reads were filtered to remove sequences shorter than 500 bp prior to assembly. Following demultiplexing, reads corresponding to each barcode were assembled independently using Flye (v2.x) with default parameters optimized for long-read sequencing, generating one draft genome per isolate. Assembly statistics for each genome are provided in Tables S4 and S5.

### Community Diversity and Composition

The Nonpareil tool 3.401,^23^ with default parameters, was used to estimate the microbial community diversity coverage for each Illumina metagenomic data set. Nonpareil essentially measures the fraction of the total extracted community DNA that was sequenced. Therefore, comparing Nonpareil sequence coverage values between metagenomes sequenced at the same sequencing effort reveals which metagenome is more diverse while taking both evenness and richness into account. Nonpareil values have been shown to generally correspond well to Shannon diversity values based on 16S rRNA gene amplicon data from the same samples and often offer higher resolution between metagenomes of similar diversity.^24^ Overall similarity between metagenomic data sets was determined based on Mash distances^25^ for the trimmed reads and visualized using a PCoA plot generated with the ggplot2 package in R.^26^ Mash compares the sequence profiles between two conditions using a k-mer frequency methodology.^25^ Permutational multivariate analysis of variance (PERMANOVA) using distance matrices was performed with the adonis function of the vegan R package.^27^

Taxonomic classification of the generated MAGs and isolate genomes was assessed with GTDB-Tk v2.3.2 against GTDB r214.^28^ The presence and relative abundance of recovered MAGs were assessed by read recruitment as described previously.^29^ Additionally, the synthetic *Escherichia coli* DEP genome (JBJXBM000000000) was used as a reference, and its presence and abundance were also assessed by read recruitment. Briefly, all metagenomic reads from each data set were mapped against individual genome sequences (MAGs or strain DEP) using stand-alone BLASTn (best match when better than 95% nucleotide identity and 70% of read length were used to identify mapped reads). Read recruitment plots were generated using scripts from the enveomics collection^30^ and visually inspected for the presence/absence of the reference genome used. For MAGs found to be present, the 80% truncated average sequencing depth (TAD80) of each MAG in each metagenomic sample (after subtracting the reads representing the spiked-in *E. coli* and normalizing to the same number of reads per sample) was subsequently estimated using CoverM 0.6.1 (https://github.com/wwood/CoverM) with the following arguments: coverm genome -p bwa-mem --min-read-percent-identity 95 --min-read-aligned-percent 75 --trim-max 90 --trim-min 10 -m trimmed_mean. TAD80 values were further normalized by the genome equivalent (GEQ) of each metagenome using MicrobeCensus^31^ to provide estimates of relative abundance that are directly comparable between samples. The GEQ normalization can account for differences in average genome size and other factors between samples, thus providing more directly comparable abundance estimates between samples.^31^ SIMPER analysis^32^ and analysis of variance (ANOVA) were used to identify statistical differences in the relative abundance of the microbial groups present in the mesocosms (T1.5) and the control. All *p* values smaller than 0.05 were considered significant.

Kraken2 was also used to assign taxonomy at the read level against a reference library that included bacteria, archaea, viruses, protozoa, humans, and fungal reference genomes^33^ for an alternative view of microbial community changes to that provided by the MAGs. The relative abundance of the Kraken2 profiles was estimated by Bracken as performed previously.^34^

### Individual Gene Detection and Estimation of Relative Abundance

For the detection and relative abundance estimation of the synthetic genes of the strain *E. coli* DEP, TAD80 values were calculated and normalized by GEQ for each gene as explained above for whole genomes. To predict the estimated theoretical limit of detection (thLOD) of the synthetic genes, the methodology previously described in ref 35 was followed under the assumption of 1.0E + 06 cells per milliliter based on our previous work with similar water samples from the river.^36^

## Results and Discussion

### Culturing Data

Four mesocosms were constructed in glass jugs filled with 12 L of water from the Chattahoochee River and sampled over time for metagenome sequencing and isolation work. The mesocosms were thoroughly stirred just before sampling, and there was no evident sedimentation, mostly because the inoculum water was clear after prefiltration, which is commonly the case for surface water from this river. Three of the mesocosms (M1, M2, and M3; biological replicates) were inoculated with the *E. coli* DEP strain, resulting in an initial concentration of 1.91E + 05 colony-forming units (CFU)/mL at the onset of the experiment, which represented about 30% of total cells in the jug (see metagenome-based abundance quantification in the next section). The fourth jug was used as a control without a DEP inoculation (C). The viable CFU counts of the *E. coli* DEP strain on permissive (BipA-supplemented) media showed that the number of viable colonies decreased by more than 50% within the first day in all three experimental mesocosms (Figure 1A), with 1.13E + 02 CFU/mL, on average, after 5 days from the start of the incubation (i.e., a 3 orders of magnitude reduction in cell counts). No colonies on permissive medium were obtained from samples from the control mesocosm.

**Figure 1:**
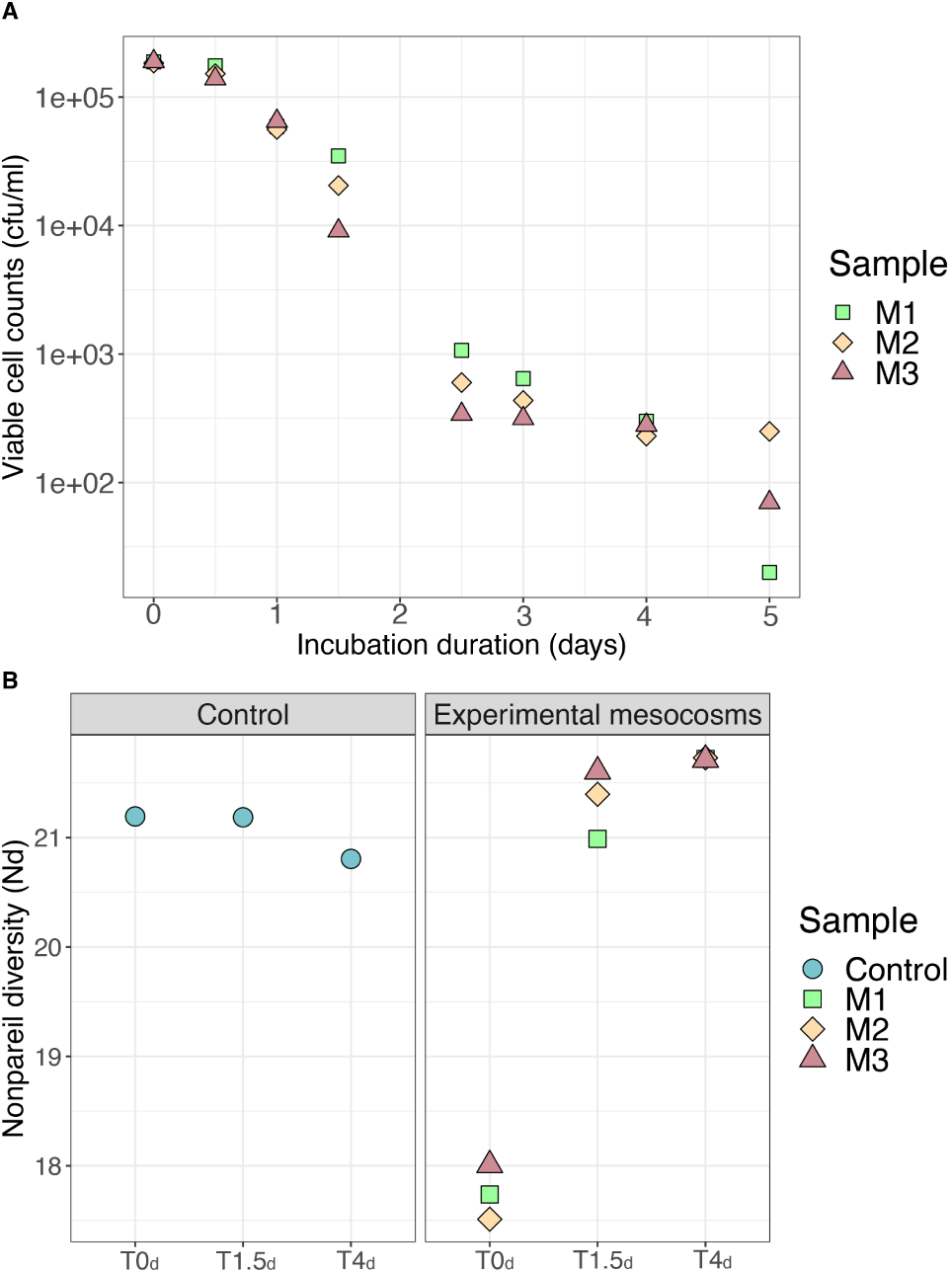
A) Decline in viable cell counts for the DEP strain as selected on chloramphenicol during the incubation period in the three experimental mesocosms (M1, M2, and M3). Note that viable DEP cells that have lost the *Cm* ^R^ plasmid are unlikely to grow (and be counted) under the conditions used. B) Nonpareil diversity (Nd) comparison of the samples used in this study (M1, M2, M3, and Control). T0_d_ indicates day 0 after inoculation, T1.5_d_ indicates day 1.5 after inoculation, and T4_d_ indicates day 4 after inoculation.

### Prokaryotic Alpha Diversity Shifts during Mesocosm Incubation

A total of 12 metagenomes were sequenced from the three experimental and one control mesocosms across three sampling time points (T0_d_, 0 days; T1.5_d_, 1.5 days; and T4_d_, 4 days), ranging in yield between 30.7 and 47.5 million Illumina short-read sequences (4.5 to 6.9 Gbp) following trimming (Table S2). For these samples, an average of 56% of the expected nucleotide diversity was recovered by our sequencing effort based on Nonpareil analysis (Table S2). The Nonpareil sequence diversity (*N*_*d*_) values, a metric of diversity that combined evenness and richness and offered comparable, if not higher, resolution than the Shannon index based on 16S rRNA gene data,^24^ showed that the diversity in the control remained relatively stable over time. In contrast, the diversity of the experimental mesocosms was initially lower at T0_d_ (Figure 1B), likely because of the high abundance of the *E. coli* DEP strain spiked in during mesocosm inoculation, and increased exponentially over time (Figure 1B). The latter increase was apparently due to the decrease in *E. coli* DEP abundance and the subsequent prevalence of the natural microbial community. This trend was consistently observed across all three experimental mesocosms (Figure 1B), revealing the high reproducibility of experimental conditions.

### Fate of the *Escherichia coli* DEP Strain in the River Mesocosm

The presence and differential abundance analysis of the synthetic *E. coli* DEP strain over time was estimated using read recruitment against its genome sequence (Accession Number: JBJXBM000000000), followed by the calculation of relative abundance by the 80% truncated average sequencing depth (TAD80) metric normalized to genome equivalents (GEQ) for comparisons across samples.^37^ Consistent with expectations, our data from both read recruitment and culture-based analyses reflected that the engineered strain was absent in the control, with the exception of a small number of reads (∼0.1× sequence depth coverage) mapping to the DEP genome from one of the control samples (T1.5_d_) which was apparently due to contamination during sample preparation for sequencing as inferred from high-identity (∼100%) matches against the DEP genome (Figure 2A). Following the addition of the engineered strain in experimental mesocosms, it represented ∼30% of the total community at T0_d_ based on metagenome read mapping, before rapidly decreasing to ∼0.04–0.1% of the total community by T4_d_ (Figure 2A). These findings were consistent and reproducible across the three experimental mesocosms. Accordingly, the observed decrease in the DEP strain’s signal in the sequence data corresponded with the culture-based results, which showed a remarkable decrease in CFU of the DEP strain on BipA-supplemented plates after about 2.5 days of inoculation. Moreover, a comparison of the rate of abundance decline of *E. coli* in silico and in situ, based on the TAD80/GEQ metric and CFU, respectively (Figure S1), showed that the metagenome-based decline would be 12% delayed relative to the viable CFU-based decline as expected due to the persistence of DNA after cell death.

**Figure 2:**
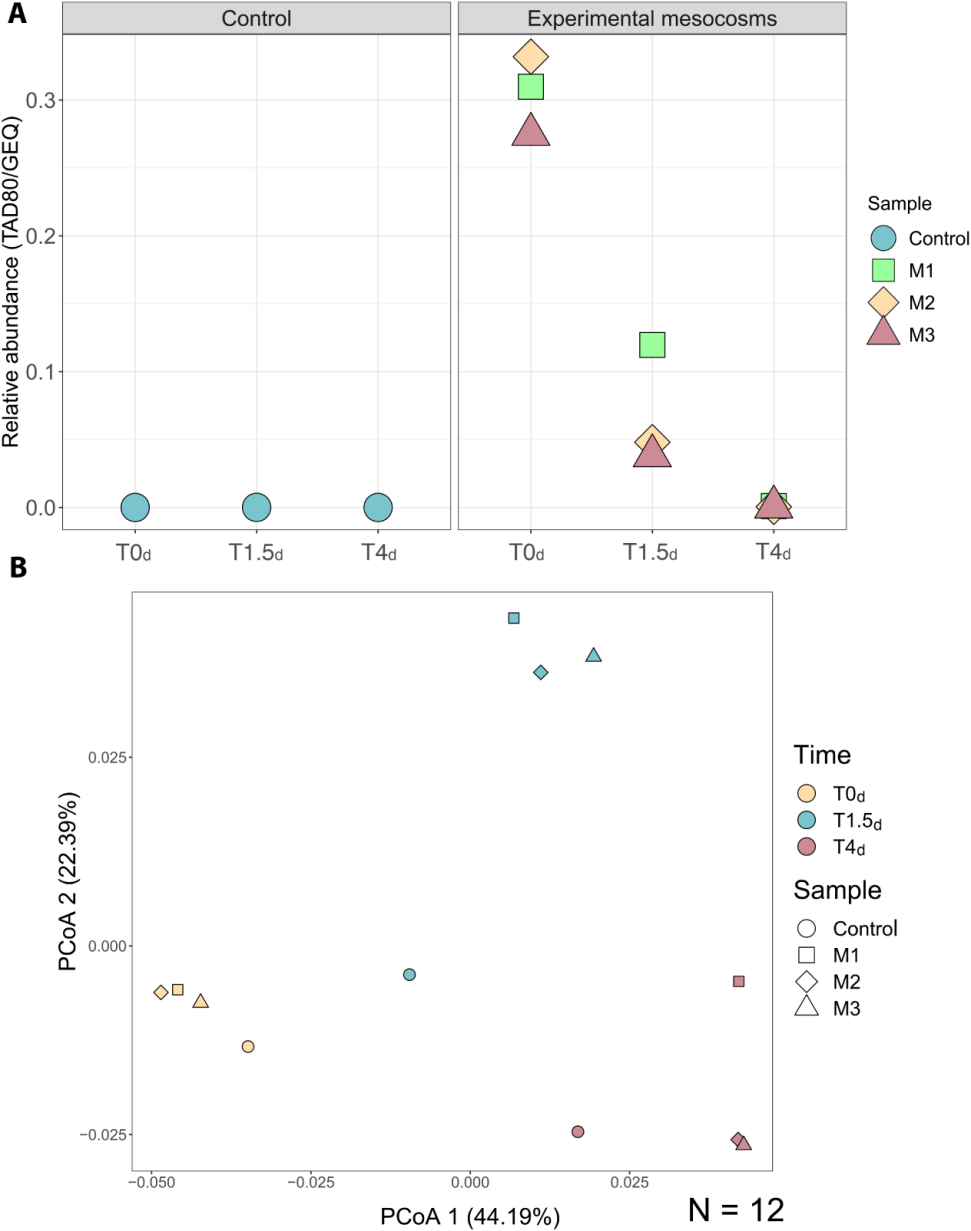
A) Relative abundance of the spike-in *E. coli* DEP strain (JBJXBM000000000) in the metagenomic data sets. Abundance was estimated based on the TAD80 metric normalized by GEQ. B) Principal coordinate analysis (PCoA) based on Mash distances showing the clustering of samples by time point (T0_d_, T1.5_d_, and T4_d_) and experiment (M1, M2, M3, and Control). Mash distances represent the overall similarity among metagenomes based on the fraction of shared kmers compared to the total kmers recovered by sequencing for each metagenome.

A few colonies were observed on the BipA-supplemented plates on day 5 of the incubation, 1.13E + 02 CFU/mL, on average (i.e., a 3 orders of magnitude reduction) within a relatively short period of time compared to the initial concentration of 1.91E + 05 CFU/mL. These colonies could indeed represent surviving DEP cells or escape events from the BipA dependence (or biocontainment). Alternatively, they could represent low-abundance, freshwater chloramphenicol-resistant bacteria that can grow on the plates autotrophically or by using BipA as a carbon source. We did not save these colonies from the day 5 plates to be able to taxonomically identify them and directly test these competing hypotheses. However, we did identify such isolates from a similar follow-up mesocosm experiment (described below), which revealed that the isolates represented environmental bacteria, consistent with the latter hypothesis and the robust biocontainment of strain DEP.

### Low Competitiveness of the *Escherichia coli* DEP Strain Relative to Freshwater Microbes

It was important to assess the extent to which the decrease in the abundance of DEP cells was due to the lack of the modified amino acid BipA in the water mesocosm vs competition and predation events with indigenous microbiota, as reported in other studies.^38^ In other words, we attempted to determine the decay kinetics of the parent strain without the requirement for the modified amino acid under the same incubation conditions. For this, we performed a new set of mesocosm incubations similar to those described above, except that the added cells were not washed, so some growth media was added to the mesocosm together with the cells to better mimic a spill event. This new set included a mesocosm spiked with cells of strain DEP (M1), one spiked with cells of strain DEP and 100 μM of BipA (M2; without arabinose, so the BipA-dependent genes on the plasmid should not be transcribed, to test for any effects of BipA and escape mutants), one spiked with cells of strain C321.ΔA_pEVOL.bipARS.d7 which is identical to strain DEP, including the presence of a chloramphenicol-resistant plasmid, but has no recoded genes that require BipA for growth (M3), and one control (M4; no cells added). [Note the different treatment applied to M2–M3 of this second set of incubations compared to M2–M3 of the first set of incubations.] Our results showed similar decay kinetics compared to the results described above, albeit slightly delayed—by 3–4 days—presumably due to the small amount of growth media present in the mesocosms, and no substantial difference in the decay of strains C321.ΔA_pEVOL.bipARS.d7 and DEP (Figure S2). These results reveal that the two strains have lost their competitive advantage in the natural environment, which is consistent with their known decreased fitness under laboratory conditions due to the mutations accumulated during the genomic recoding process,^39^ although their fitness can be recovered based on adaptive laboratory evolution (Kunjapur et al., unpublished). In any case, our results collectively show that strain DEP does not proliferate in our freshwater mesocosms in the absence of BipA and has minimal impacts on the indigenous microbial communities, indicating low environmental risk from an accidental release of this specific SynBio strain.

Similar to the first set of mesocosms described above, we observed a few colonies at day 5 or later sampling points on permissive media (≤1.13E + 02 CFU/ml) from mesocosms M1–M3, but not the control mesocosm. Sequencing of 21 of these isolates using an in-house Oxford Nanopore sequencer, chosen to represent the diversity of colony morphologies observed on the permissive (BipA-supplemented) or nonpermissive (no BipA added) culturing plates, revealed that the great majority of these isolates (17/21) were environmental bacteria of the *Pseudomonas* and *Aeromonas* genera (Tables S4 and S5). These isolates encoded several antibiotic resistance genes in their genome (e.g., efflux pumps) to cope with the chloramphenicol present in the media but not the chloramphenicol-resistant gene present in the DEP strain, indicating no HGT from DEP. The remaining four isolates were identified as *E. coli* with high identity (>99.99 ANI) to the DEP genome, and represented the DEP strain recovered on permissive media (n = 1) and strain C321.ΔA_pEVOL.bipARS.d7 recovered on nonpermissive media (n = 3). These results indicated that a few *E. coli* isolates survived the incubation period apparently based on the residual growth media added to the mesocosm together with the cells. Consistently, no major mutations were observed in the genome of these *E. coli* isolates, including the plasmid carrying the BipA-dependent genes, indicating that they are unlikely to represent escape events. While it was not completely clear why we obtained *Pseudomonas* spp. and *Aeromonas* spp. isolates only from the treated (spiked) mesocosms and not the control, we noticed an increase in the abundance of these taxa over time in the metagenomes from the treated mesocosms of the first incubation set, emerging from the rare biosphere based on read recruitment (Figure S3) but not becoming abundant enough for their genomes to be recovered as a MAG. Accordingly, it appears as if these organisms grew as an effect of the spiked *E. coli* cells, possibly feeding on dead cells or residual media, and thus it is easier to isolate them at later time points from the treated mesocosms (but not the control) since we use only 50 μL from each mesocosm at each sampling time point for cultivation. In any case, our results collectively suggested the low competitiveness of strain DEP as the main reason underlying its fast decay in the mesocosms and revealed no HGT of BipA-dependent genes or mutations that could have caused the escape of the biocontainment strategy. These findings are also consistent with previous reports on low frequencies of escape events for this type of SynBio organism.^8,9^

### Community Structure and SynBio’s Effects at the Whole Community Level

Analysis of beta diversity revealed a clear clustering of the metagenomes by time point (p-value: 0.001) rather than by experiment (p-value: 0.88), regardless of the *E. coli* spike-in, and especially at the last sampling time point (day 4), where the abundance and effect of the *E. coli* spike-in cells were largely decreased (Figure 2B). These results indicated that the overall microbial community was not impacted much by the *E. coli* addition; otherwise, we would have seen a clearer clustering of the samples from the experimental mesocosms, separating them from the control samples. The results also indicated that the “bottle effect” from the incubations^40^ had a much greater effect on overall community similarity than the *E. coli* addition and presumably accounted for the clustering of the control samples together with the experimental mesocosm samples. This hypothesis is consistent with other studies in which the “bottle effect” of confinement also impacts microbial communities.^41^

To explore in detail any finer impacts of the added *E. coli* strain on the community, the relative abundance of the recovered MAGs from all metagenomes was determined and assessed for any differences that correlated with the abundance of the *E. coli* strain. Our data showed that there were a few MAGs whose relative abundance significantly increased in the experimental mesocosms (T1.5_d_) in comparison to the control (e.g., g__CAMCTV01, f__UBA4408, g__UBA952, and *Fluviicola* sp.) (Figure 3). None of these MAGs could be assigned to any previously described species (Table S3), and thus might represent novel taxa to be named. More specifically, the relative abundance of *Fluviicola* sp. increased from ∼0.1% (control) to ∼0.6% (mesocosm, T1.5_d_) (p-values: 0.001 and 0.04 for SIMPER and ANOVA tests, respectively), g__CAMCTV01 from ∼0.5% (control) to ∼7% (mesocosm, T1.5_d_) (p-values: 0.001 and 0.001), f__UBA4408 from ∼0.1% (control) to ∼1.5% (mesocosm, T1.5_d_) (p-values: 0.001 and 0.0002), and g__UBA952 from ∼0.1% (control) to ∼0.9% (mesocosm, T1.5_d_) (p-values: 0.001 and 0.002). These MAGs might have benefited from the spike-in of *E. coli* cells in the community, probably due to favorable conditions, including bottle effects, caused by the incubation, or—possibly—feeding upon the dying *E. coli* cells. These findings indicated that while the community was not impacted much by the spike-in *E. coli* cells, a few specific groups might have been affected, mostly positively. To further corroborate these findings, the overall community taxonomic composition of the generated data sets was characterized at the (unassembled) read level using Kraken2 and Bracken. These results were consistent with those mentioned above showing rather limited effects of the inoculation and further corroborating the abovementioned conclusions (Figure S4).

**Figure 3:**
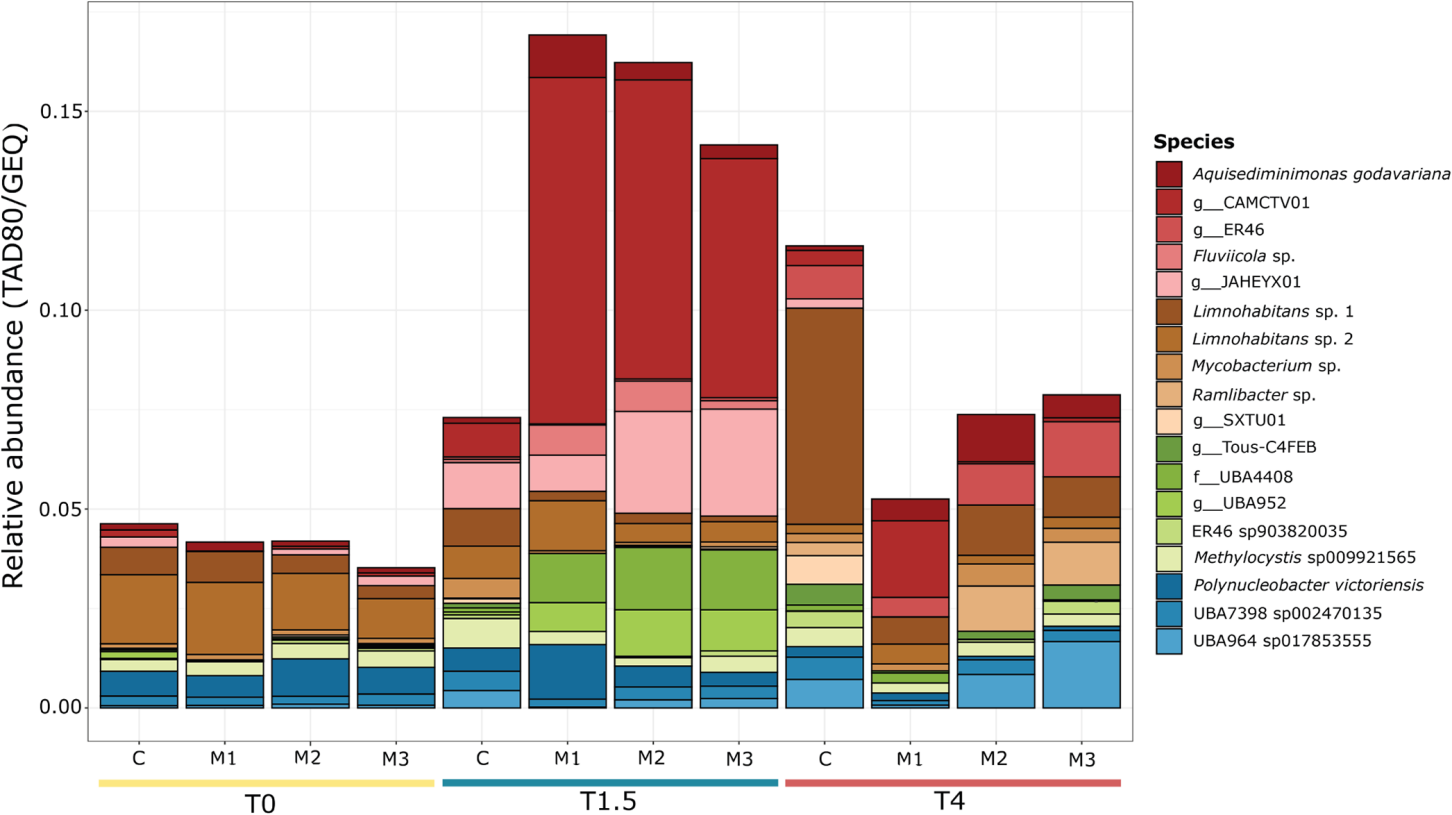
Relative abundance of the recovered MAGs across samples. Relative abundance was estimated as the TAD80 metric normalized by GEQ (see Materials and Methods for details). The *E. coli* MAG (strain DEP population) is not shown to avoid changing the scale (and thus the resolution) of the *y*-axis due to its very high abundance at T0_d_ (please see Figure 2 instead for this MAG). For MAGs that cannot be assigned to a known species, their closest relative available in GTDB, denoted by an alphanumeric identifier, is shown instead, including the lowest classification shared with the relative based on RED values, e.g., “g” for genus-level assignment and “f” for family-level assignment.

### Absence of HGT Events of Recoded Genes to Indigenous Community Members

In addition to the isolate genome-based results reported above, we screened for HGT transfer events of the modified genes (*adk.d6*, *bipARS*, *tyrS.d8,* and *Cm* ^R^) from the SynBio organism to members of the natural microbial community based on the relative abundance of the genes relative to the *Escherichia coli* genome using read recruitment. Our data showed that the relative abundance of the *adk.d6* and *tyrS.d8* genes decreased in the mesocosms at the same rate as the *E. coli* DEP strain (Figure 4), indicating the absence of HGT events of these genes to other members of the freshwater microbial community (otherwise, a higher abundance of the genes relative to the genome would have been observed). The relative abundance of *bipARS* and *Cm* ^R^ markers followed a pattern identical to that of *E. coli* DEP (Figure 4), albeit the abundance of these genes was higher than the *adk.d6* and *tyrS.d8* genes or the whole genome due to their presence in a high-copy plasmid. In other words, we observed a consistent gene copy number between modified genes and the genome copy number of the engineered strain at each sampling point during mesocosm operation. Additionally, the four modified genes were searched for within the MAGs recovered from the mesocosms. Our results showed that the *adk.d6* and *tyrS.d8* genes were only found in the MAG taxonomically assigned as *E. coli*, further corroborating the absence of HGT events of these genes during our mesocosm incubation. It is worth mentioning that, given the inherently low sensitivity of metagenomics for detecting rare HGT events,^42^ it is possible that some low-frequency transfers might have happened (and were not detected by our approach). The average estimated theoretical limit of detection (thLOD) in our metagenomic data set was estimated to be 7.55E + 01 ± 1.76E + 01 (cells/mL) (for key assumptions, see the Materials and Methods section). Therefore, HGT events of the modified genes, if present in the experimental mesocosms, should be at abundances lower than 7.55E + 01 ± 1.76E + 01 (cells/mL). Despite this limitation, however, the relatively high diversity coverage obtained by our sequencing effort (56%),^43^ the rigorous data analysis performed, and the absence of HGT of the *adk.d6* and *tyrS.d8* genes based on the isolate genome sequencing mentioned above consistently supported that major HGT events involving the DEP strain were absent.

**Figure 4:**
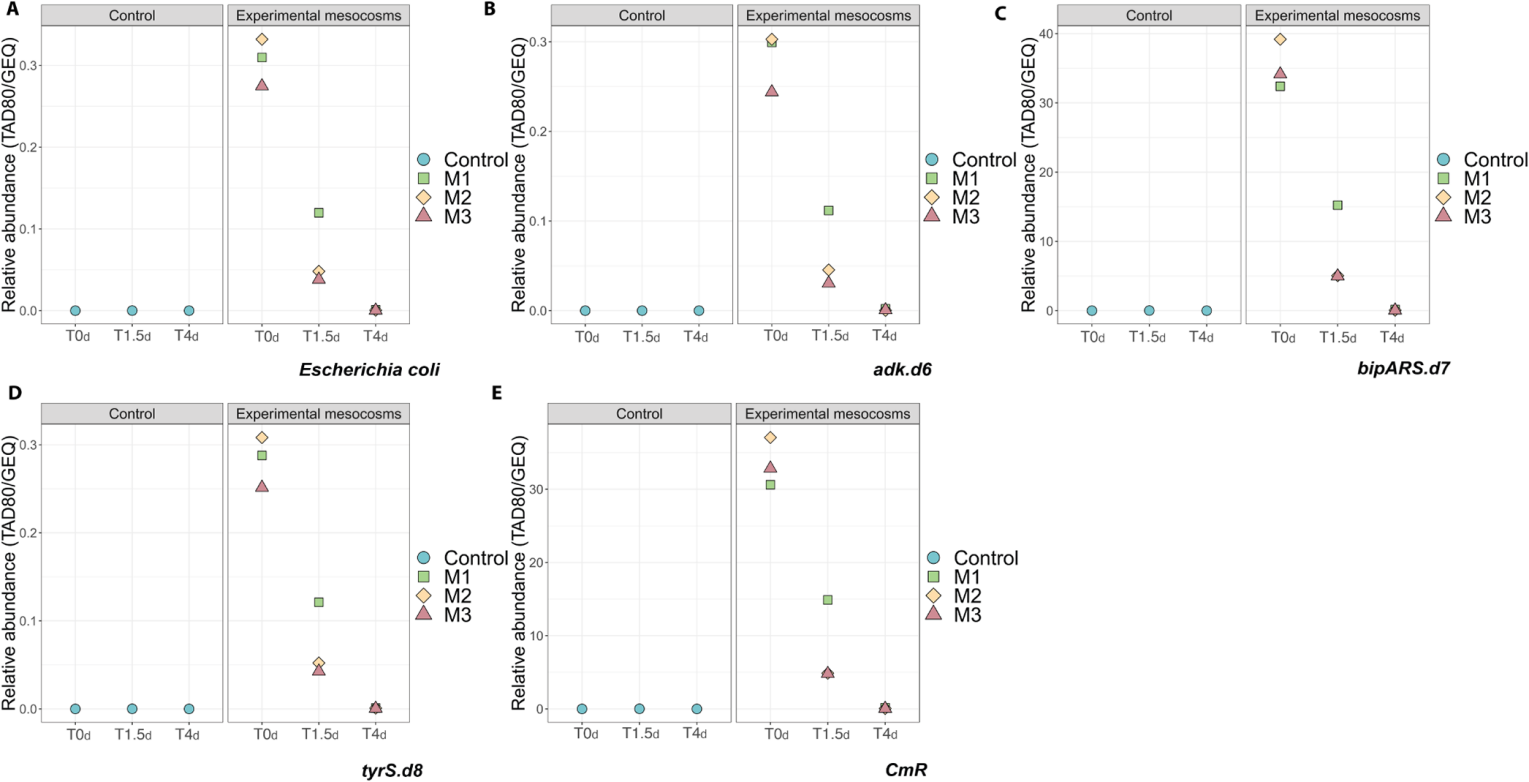
Relative abundance of (A) *E. coli* DEP strain and the modified genes (B) *adk.d6,* (C) *bipARS,* (D) *tyrS.d8*, and (E) *Cm* ^R^) in the metagenomic data sets. The strain data in panel A is identical to those in Figure 2A but are provided here as a reference point for comparison to the gene graphs shown in the same panel.

## Concluding Remarks

Comprehensive reviews in the field emphasize that although engineered containment systems perform robustly in laboratory conditions, the real-world environmental context (e.g., variable nutrients, microbial community interactions, open ecosystems) remains an under-studied domain.^44,45^ Our findings from laboratory incubations that closely mimic the natural freshwater environment collectively support that the SynBio *Escherichia coli* DEP strain may not represent a serious environmental threat or risk upon its accidental release into the environment primarily due to its low competitiveness in the natural environment. Our results also show that the biocontainment strategy for strain DEP is robust because the genetic alterations introduced into the strain are not easily transferred nor do they introduce new traits into the natural microbial communities or drastically alter community composition. Consistent with these expectations, previous observations suggest effective biocontainment of *E. coli* DEP strain after 14 days in other settings, underscoring its long-term stability.^9^ Further, other studies have shown that genome-recoded organisms with dependence on noncanonical amino acids show escape frequencies in the <10^–10^ range.^8,46^ Dependence on noncanonical amino acids for several genes, as is the case for strain DEP, can also reduce the importance of horizontal gene transfer as an escape mechanism because recombination of multiple auxotrophic genes concomitantly (with their nonauxotrophic homologues to escape auxotrophy) is highly unlikely,^8,46^ consistent with the results observed here showing no escape or horizontal gene transfer events (e.g., Figure 4 and isolation work).

The use of both sequencing and culture-based assays herein to track the persistence and fate of *E. coli* DEP enabled a holistic assessment of biosafety concerns, addressing some of the limitations of (only) culture-based approaches, as recommended previously,^47,48^ and thus is recommended for future biosafety-related studies of SynBio organisms. Notably, this same methodology, with some adjustments to account for the sequences representing the genetic modifications in question, can be used for studying other recoded strains. It is also important to emphasize that while strain DEP’s competitiveness in the natural environment is apparently low based on our metagenome- and isolate-based results, and this largely accounts for its fast decay kinetics, such fast decay may not be the case for other SynBio organisms, including those encoding noncanonical amino acids in their genomes.

## Supplementary Material



## Data Availability

Raw sequence reads from each metagenome are publicly available in the SRA database under BioProject PRJNA1187556.

## #

Department of Environmental & Energy Engineering, The University of Suwon, 17, Wauan-gil, Bongdam-eup, Hwaseong-si, Gyeonggi-do, 18323, South Korea

## ∇

Department of Chemical Engineering and Applied Chemistry, University of Toronto, Toronto, ON, M5S 3E5, Canada

## ⬢

Facultad de Salud y Deporte, Universidad Alfonso X el Sabio – Mare Nostrum, Camino de la Térmica, 44, 29004 , Málaga, España
