# Supplementary material for: A Metagenome-Based Methodology to Track Genomically Recoded Strains and Assess Their Effects on Indigenous Microbes
Source: Environ Sci Technol. 2026 Jul 20;60(30):21061–70. doi: 10.1021/acs.est.5c15663 (PMC13449894; doi:10.1021/acs.est.5c15663)
Supplement: Supplementary file 1 [file es5c15663_si_001.pdf]

Supplementary Material:

A metagenome-based methodology to track genomically recoded strains and assess their effects on indigenous microbes.

Ana Durán-Viseras[1 #], Gyuhyon Cha[1, †], Janet K. Hatt[1], Blake G. Lindner[1, ‡], Efterpi M. Benvenuto[1], Yiran Zhang[1], Aditya M. Kunjapur[2], Konstantinos T. Konstantinidis[1,*]

[1]School of Civil and Environmental Engineering, Georgia Institute of Technology, Atlanta, GA 30332, USA

[2]Chemical and Biomolecular Engineering, University of Delaware, Newark, DE 19716, USA

[#] Current address: Facultad de Salud y Deporte, Universidad Alfonso X el Sabio – Mare Nostrum, Camino de la Térmica, 44, 29004 , Málaga, España

†Current address: Department of Environmental & Energy Engineering, The University of Suwon, 17, Wauan-gil, Bongdam-eup, Hwaseong-si, Gyeonggi-do, 18323, South Korea

[‡]Current address: Department of Chemical Engineering and Applied Chemistry, University of Toronto, Toronto, ON, M5S 3E5, Canada

*Corresponding author; email: kostas@ce.gatech.edu

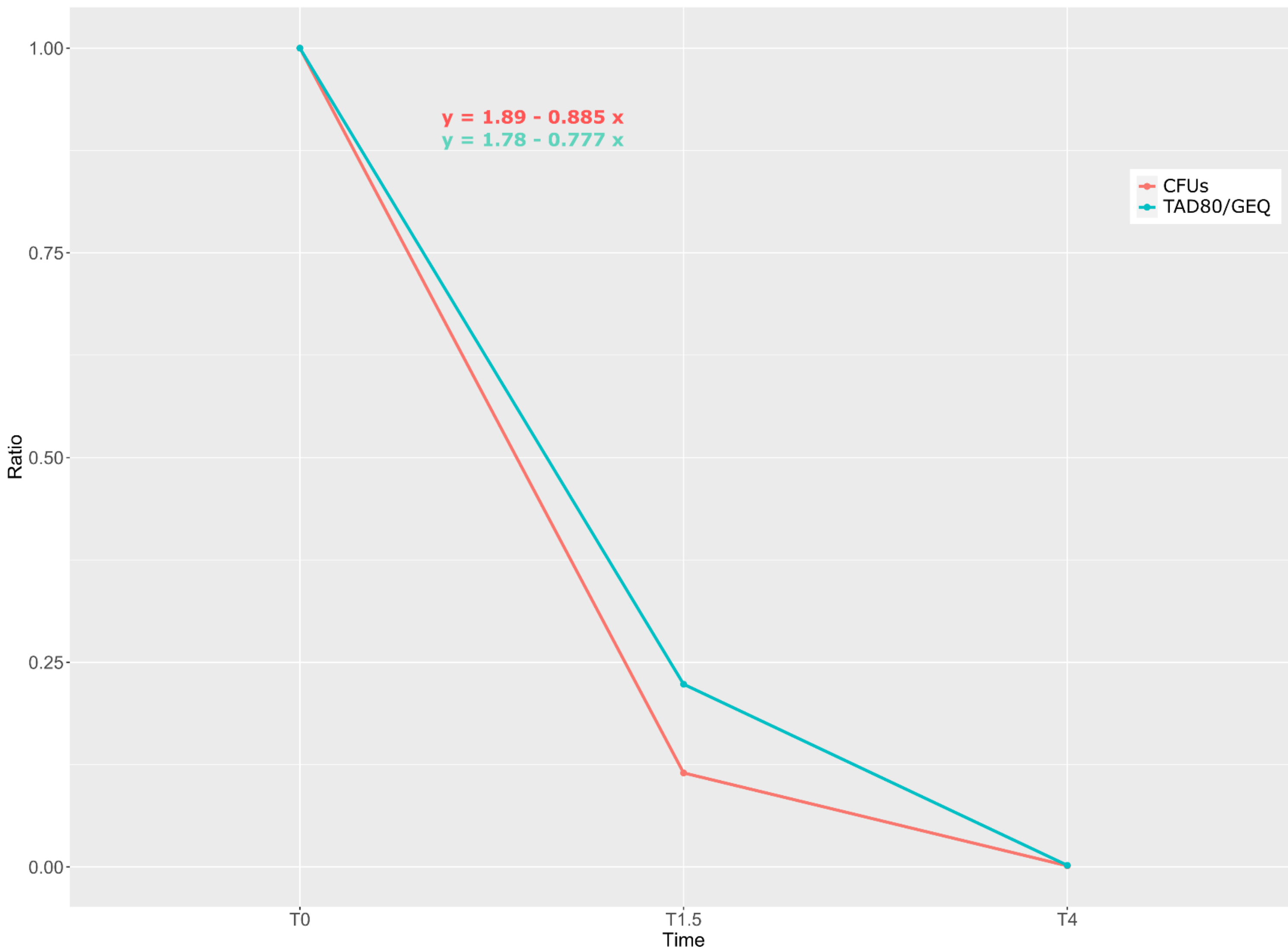

Figure S1. Representation of the decline in the relative abundance rate of *Escherichia coli* in the first set of mesocosm incubations over time, based on the TAD80/GEQ estimation and CFU counts.

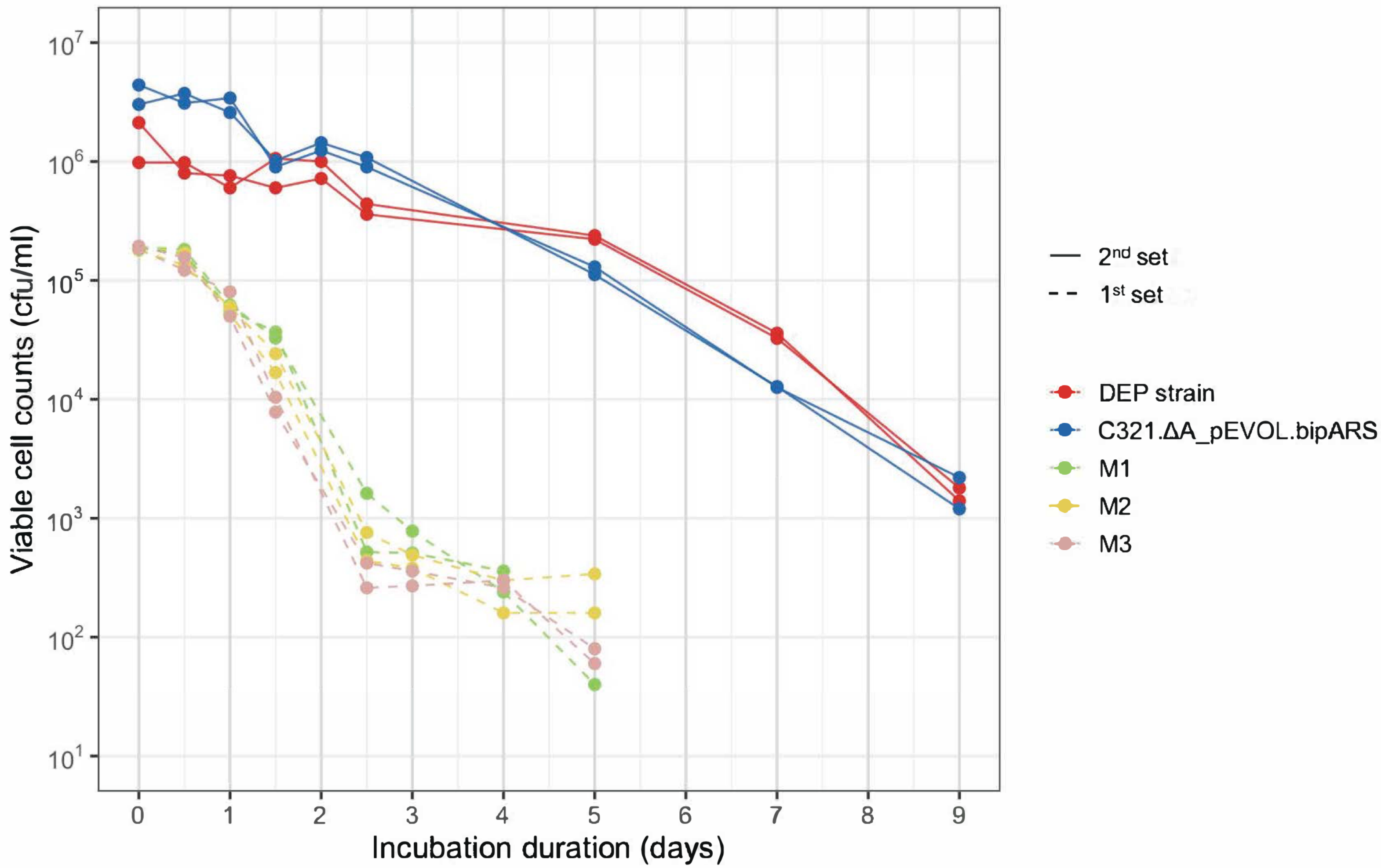

Figure S2. Viable cell counts (CFU/ml) on permissive media for the DEP strain and C321.ΔA_pEVOL.bipARS from time-resolved samples of the 2nd mesocosm set (in red and blue) compared to control mesocosms and data from the 1st mesocosm set (M1–M3). Each point represents an individual mesocosm replicate, and lines connect measurements from the same replicate. Experimental data from the 2nd set (i.e., cells added without washing, so some residual permissive growth media were added to the mesocosm) are shown as solid lines, while data from the 1st set of incubations are shown as dashed lines. The y-axis is displayed on a logarithmic scale. No growth was detected in the control mesocosm at any time point (hence, no line representing control is included).

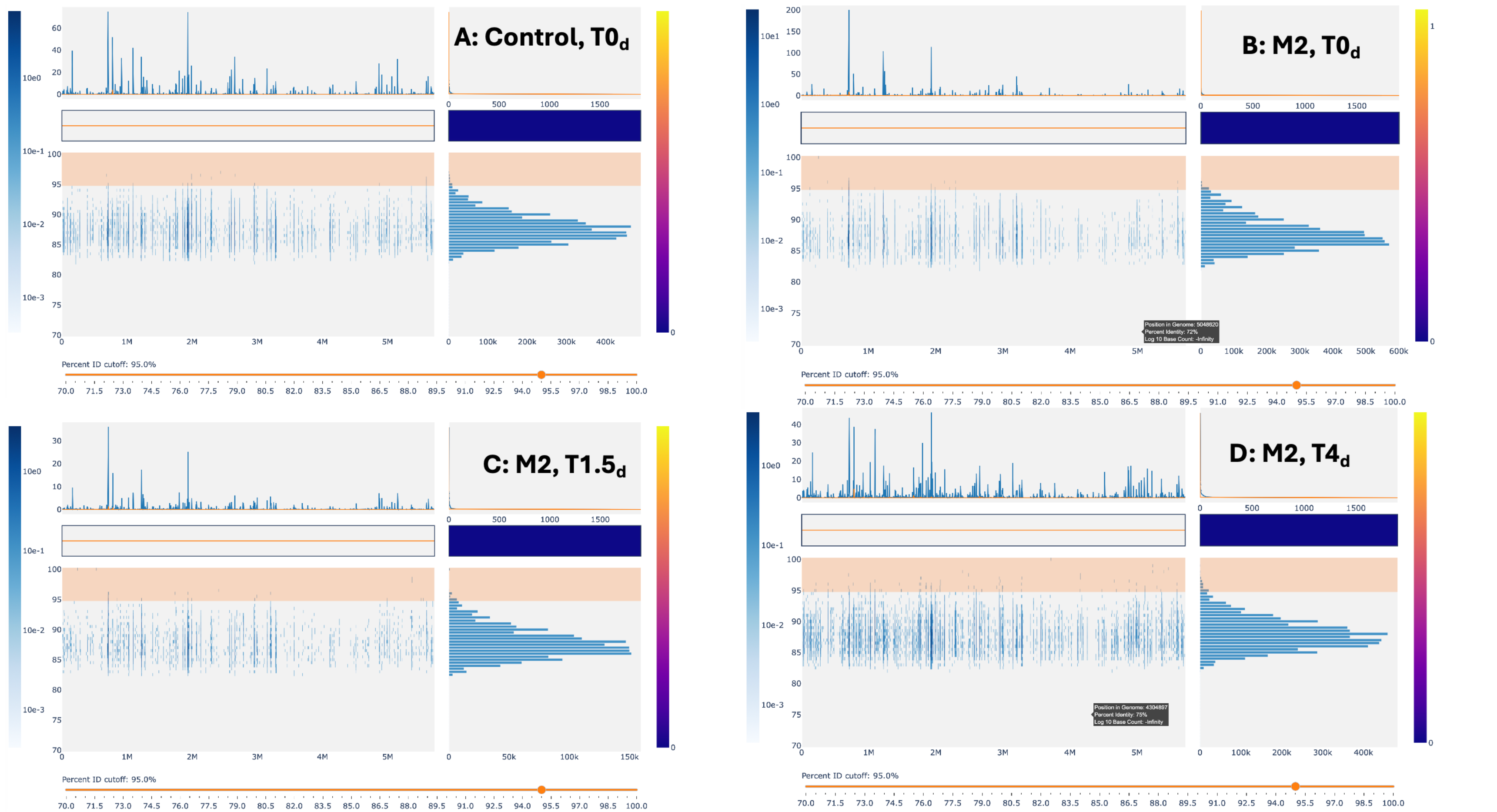

Figure S3. Read recruitment plot for the *Pseudomonas thermotolerans* isolate (barcode20) against metagenomic datasets from M2 and control mesocosms of the first incubation set. Illumina metagenomic reads were mapped to the isolate genome using bwa_mem, and alignments were filtered to retain high confidence matches only (mapping quality ≥20 and aligned length ≥100 bp). Each point represents a read mapped along the genome (x-axis), with the y-axis indicating percent nucleotide identity. The upper panel shows sequencing depth across the genome, and the right panel summarizes read identity distribution as previously described (Gerhardt et al., Frontiers in Bioinformatics, 2021). Note that the frequency of reads, especially those mapping to the reference genome with at least 95% nucleotide identity, increases over time in M2, particularly in $T4_d$. In contrast, control samples show consistently lower read frequency. Note that reads mapping in the 85–95% range, representing close relatives of the reference, also increase with time.

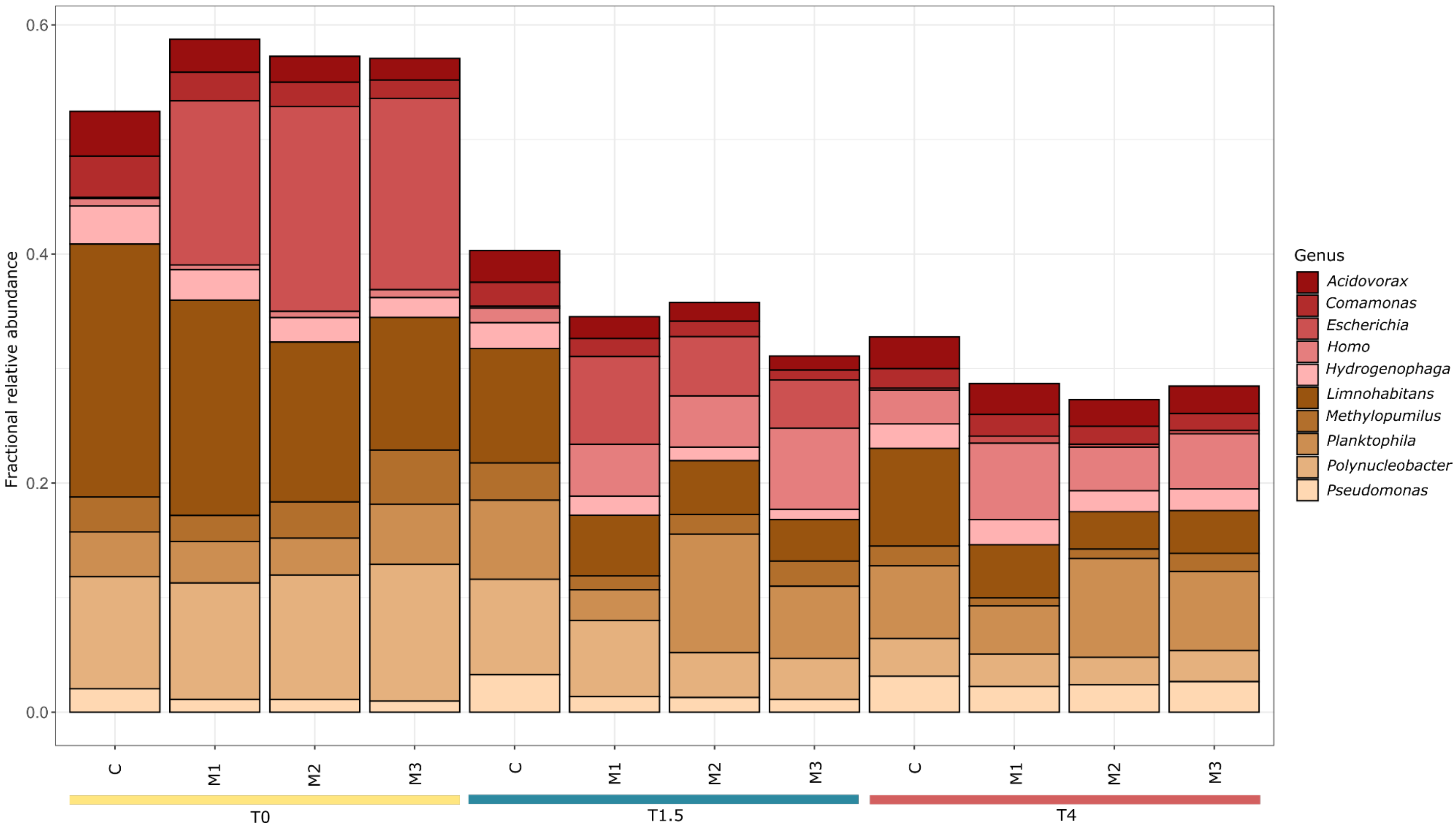

Figure S4. Average relative abundance of the top ten most abundant genera (according to Kraken) in the mesocosms. The underlying data are metagenomic short reads annotated by Kraken/Bracken.

Table S1. Viable cell counts (in CFU) for the DEP strain on chloramphenicol-supplemented media during the incubation period in the three mesocosms (M1, M2 and M3) of the first set of incubations.

Time	Sample	CFUs
0	M1	1.90E+05
0	M1	1.86E+05
0.5	M1	1.82E+05
0.5	M1	1.68E+05
1	M1	6.20E+04
1	M1	5.60E+04
1.5	M1	3.28E+04
1.5	M1	3.68E+04
2.5	M1	1.62E+03
2.5	M1	5.20E+02
3	M1	7.80E+02
3	M1	5.10E+02
4	M1	2.40E+02
4	M1	3.60E+02
5	M1	4.00E+01
5	M1	0.00E+00
0	M2	1.88E+05
0	M2	1.80E+05
0.5	M2	1.70E+05
0.5	M2	1.34E+05
1	M2	5.20E+04
1	M2	6.00E+04
1.5	M2	1.68E+04
1.5	M2	2.42E+04
2.5	M2	4.40E+02

2.5	M2	7.60E+02
3	M2	3.80E+02
3	M2	4.90E+02
4	M2	1.60E+02
4	M2	3.00E+02
5	M2	1.60E+02
5	M2	3.40E+02
0	M3	1.84E+05
0	M3	1.94E+05
0.5	M3	1.22E+05
0.5	M3	1.56E+05
1	M3	8.00E+04
1	M3	5.00E+04
1.5	M3	1.04E+04
1.5	M3	7.80E+03
2.5	M3	2.60E+02
2.5	M3	4.20E+02
3	M3	2.70E+02
3	M3	3.60E+02
4	M3	3.00E+02
4	M3	2.60E+02
5	M3	6.00E+01
5	M3	8.00E+01

Table S2. Statistics of the 12 metagenomic datasets analyzed in this study from samples of the first set of incubations.

Sample	Experiment	Time	Reads			
			Total reads	Total bp	Nonpareil coverage	Nonpareil diversity
C-T0_S1	Control	0 days (T0)	33580194	4.90E+09	0.5151036	21.1936
C-T1-5_S5	Control	1.5 days (T1.5)	32747396	4.75E+09	0.51559	21.18541
C-T4C_S9	Control	4 days (T4)	47509256	6.89E+09	0.6053539	20.80568
M1-T0_S2	M1	0 days (T0)	32220238	4.71E+09	0.7403951	17.73731
M1-T1-5_S6	M1	1.5 days (T1.5)	41007428	5.92E+09	0.5494003	20.988
M1-T4C_S10	M1	4 days (T4)	41409470	5.95E+09	0.4719659	21.71936
M2-T0_S3	M2	0 days (T0)	32396122	4.70E+09	0.740837	17.51168
M2-T1-5_S7	M2	1.5 days (T1.5)	30701316	4.46E+09	0.4671178	21.396
M2-T4C_S11	M2	4 days (T4)	45465794	6.60E+09	0.4803949	21.72925
M3-T0_S4	M3	0 days (T0)	36204350	5.27E+09	0.7177103	18.00636
M3-T1-5_S8	M3	1.5 days (T1.5)	37389162	5.41E+09	0.4620585	21.60464
M3-T4C_S12	M3	4 days (T4)	45707872	6.61E+09	0.4822455	21.71102

Table S3. Statistics and taxonomy assignment of the MAGs obtained in this study from samples of the first set of incubations.

MAG	No. contigs	Completeness (%)	Contamination (%)	Strain Heterogeneity	Length (bp)	GC (%)	N50 (bp)	GTDB_Taxonomy
maxbin_IDBA_norm_M2-T0_S3.005	383	82,52	19,66	60,71	3193703	58,93	8165	d__Bacteria;p__Pseudomonadota;c__Gammaproteobacteria;o__Burkholderiales;f__Burkholderiaceae_B;g__Limnohabitans;s__
maxbin_metaSpades_norm_C-T4C_S9.009	132	75,86	13,01	0	1451105	44,06	12517	d__Bacteria;p__Actinomycetota;c__Actinomycetia;o__Nanopelagicales;f__Nanopelagicaceae;g__UBA7398;s__UBA7398 sp002470135
metabat_IDBA_C-T1-5_S5.bin.35	104	75,51	7,52	89,47	1621086	49,14	19235	d__Bacteria;p__Pseudomonadota;c__Alphaproteobacteria;o__Rhizobiales;f__Beijerinckiaceae;g__Methylocystis;s__Methylocystis sp009921565
metabat_IDBA_C-T4C_S9.bin.12	172	92,08	0	0	2795862	61,04	20905	d__Bacteria;p__Verrucomicrobiota;c__Verrucomicrobiae;o__Opitutales;f__Opitutaceae;g__Tous-C4FEB;s__
metabat_IDBA_M1-T1-5_S6.bin.1	178	81,93	0,58	100	2530910	64,07	17258	d__Bacteria;p__Pseudomonadota;c__Alphaproteobacteria;o__Sphingomonadales;f__Sphingomonadaceae;g__Aquisediminimonas;s__Aquisediminimonas godavariana
metabat_IDBA_M1-T1-5_S6.bin.7	185	79,01	0	0	2287152	38,23	14476	d__Bacteria;p__Bacteroidota;c__Bacteroidia;o__Flavobacteriales;f__Crocinitomicaceae;g__Fluviicola;s__
metabat_IDBA_M2-T4C_S11.bin.18	167	92,39	1,13	20	3093582	65,03	22866	d__Bacteria;p__Actinomycetota;c__Actinomycetia;o__Mycobacteriales;f__Mycobacteriaceae;g__Mycobacterium;s__
metabat_IDBA_M3-T1-5_S8.bin.8	147	95,71	0,48	0	2973576	40,27	24570	d__Bacteria;p__Bacteroidota;c__Bacteroidia;o__AKYH767;f__UBA4408;g__;s__
metabat_IDBA_M3-T4C_S12.bin.2	325	79,49	0,71	100	3519391	68,24	11374	d__Bacteria;p__Verrucomicrobiota;c__Verrucomicrobiae;o__Opitutales;f__Opitutaceae;g__ER46;s__
metabat_IDBA_norm_C-T1-5_S5.bin.13	108	88,92	0	0	2238065	39,97	29656	d__Bacteria;p__Bacteroidota;c__Bacteroidia;o__Chitinophagales;f__CAIODY01;g__JAHEYX01;s__
metabat_IDBA_norm_M1-T1-5_S6.bin.22	40	91,92	0,4	50	1655765	42,8	60877	d__Bacteria;p__Pseudomonadota;c__Gammaproteobacteria;o__Burkholderiales;f__Burkholderiaceae;g__Polynucleobacter;s__Polynucleobacter victoriensis
metabat_IDBA_norm_M3-T4C_S12.bin.9	47	94,47	1,49	0	2416284	64,53	89132	d__Bacteria;p__Pseudomonadota;c__Gammaproteobacteria;o__Steroidobacterales;f__Steroidobacteraceae;g__UBA964;s__UBA964 sp017853555
metabat_metaSpades_C-T4C_S9.bin.11	173	96,62	1,24	0	5660530	61,92	50427	d__Bacteria;p__Verrucomicrobiota;c__Verrucomicrobiae;o__Pedosphaerales;f__SXTU01;g__SXTU01;s__
metabat_metaSpades_C-T4C_S9.bin.9	462	80,82	18,15	9,68	6092774	65,36	16711	d__Bacteria;p__Verrucomicrobiota;c__Verrucomicrobiae;o__Opitutales;f__Opitutaceae;g__ER46;s__ER46 sp903820035
metabat_metaSpades_M1-T4C_S10.bin.27	28	100	0	0	2560118	42,75	210006	d__Bacteria;p__Bacteroidota;c__Bacteroidia;o__Flavobacteriales;f__UA16;g__CAMCTV01;s__
metabat_metaSpades_M2-T1-5_S7.bin.18	63	100	0,54	0	2350953	44,69	55345	d__Bacteria;p__Bacteroidota;c__Bacteroidia;o__Flavobacteriales;f__Crocinitomicaceae;g__UBA952;s__
metabat_metaSpades_norm_M1-T0_S2.bin.1	48	98,59	0,04	0	4486435	50,75	152713	d__Bacteria;p__Pseudomonadota;c__Gammaproteobacteria;o__Enterobacterales;f__Enterobacteriaceae;g__Escherichia;s__Escherichia coli
metabat_metaSpades_norm_M2-T4C_S11.bin.6	13	99,61	0,08	0	2932928	57,29	286195	d__Bacteria;p__Pseudomonadota;c__Gammaproteobacteria;o__Burkholderiales;f__Burkholderiaceae_B;g__Limnohabitans;s__
metabat_metaSpades_norm_M3-T4C_S12.bin.10	57	99,84	0,03	0	3847065	68,94	133336	d__Bacteria;p__Pseudomonadota;c__Gammaproteobacteria;o__Burkholderiales;f__Burkholderiaceae_B;g__Ramlibacter;s__

Table S4. Taxonomic classification of isolates recovered from the second set of mesocosms (M1–M3) at late incubation time points (days 9, 12, and 20.5). Isolates were classified using both MiGA and GTDB-Tk, with consistent results observed between methods. GTDB-Tk classifications and corresponding ANI (%) values to the best match (3rd column) are reported. For media:
Permissive: LB agar plates (1.5% w/v) supplemented with chloramphenicol (34 µg/mL), L-arabinose (0.2% w/v), SDS (0.005% w/v), Tris–HCl (20 mM, pH 7.5), and BipA (100µM). These conditions provide all required components for growth of the DEP strain.
Non-permissive: LB agar plates (1.5% w/v) supplemented with chloramphenicol (34 µg/mL), L-arabinose (0.2% w/v), SDS (0.005% w/v), Tris–HCl (20 mM, pH 7.5), but lacking BipA. These conditions are non-permissive for the DEP strain and were used to identify potential escape mutants or BipA-independent growth.
Cm-only:LB agar plates (1.5% w/v) supplemented with chloramphenicol only (34 µg/mL). These conditions select for organisms with intrinsic chloramphenicol resistance and do not support DEP growth due to the absence of BipA and induction components.

Mesososm	Incubation duration (days)	Species	ANI %	Barcode number	Media recovered on
**M1**	9	*Aeromonas caviae*	97.73	3	non-permissive
**M1**	9	*Pseudomonas nicosulfuronedens*	92.75	21	cm-only
**M1**	12	*Escherichia coli* DEP strain	99.96	4	permissive
**M1**	12	*Pseudomonas nicosulfuronedens*	92.93	5	permissive
**M1**	12	*Aeromonas caviae*	97.85	23	non-permissive
**M1**	20.5	*Pseudomonas peradeniyensis*	96.32	12	permissive
**M2**	9	*Pseudomonas peradeniyensis*	96.78	17	permissive
**M2**	9	*Pseudomonas mosselii*	99.17	18	non-permissive
**M2**	9	*Aeromonas caviae*	97.9	19	cm-only

**M2**	9	*Pseudomonas thermotolerans*	97.73	20	cm-only
**M2**	12	*Aeromonas caviae*	97.73	6	permissive
**M2**	12	*Aeromonas caviae*	97.86	7	non-permissive
**M2**	12	*Pseudomonas peradeniyensis*	96.83	8	non-permissive
**M2**	12	*Pseudomonas shirazica*	97.42	9	non-permissive
**M2**	12	*Aeromonas caviae*	97.88	10	cm-only
**M2**	12	*Pseudomonas peradeniyensis*	96.79	22	permissive
**M2**	20.5	*Pseudomonas mosselii*	95.66	13	permissive
**M3**	12	*Escherichia coli* C321.ΔA pEVOL-BipARS.d7	99.97	14	non-permissive
**M3**	20.5	*Escherichia coli* C321.ΔA pEVOL-BipARS.d7	99.97	15	cm-only
**M3**	20.5	*Escherichia coli* C321.ΔA pEVOL-BipARS.d7	99.96	24	cm-only
**M3**	20.5	*Pseudomonas kurunegalensis*	96.81	16	cm-only

Table S5. Summary statistics of isolate genome assemblies. Reported metrics include genome size (bp), N50 (bp), number of contigs, GC content (%), completeness (%), and contamination (%).

Isolate	Genome size (bp)	N50 (bp)	Contigs	GC (%)	Completeness (%)	Contamination (%)
**barcode03**	4,792,371	4,792,371	1	61	100	0.84
**barcode04**	4,670,677	4,647,543	9	51	100	0.14
**barcode05**	6,578,028	6,578,028	1	65	100	0.02
**barcode06**	4,794,021	4,794,021	1	61	100	0.85
**barcode07**	4,792,722	4,792,722	1	61	100	0.85
**barcode08**	6,038,335	6,038,335	1	65	100	0.02
**barcode09**	5,550,660	5,550,660	1	63	100	0.04
**barcode10**	4,793,793	4,793,793	1	61	100	0.84
**barcode12**	6,056,075	6,056,075	1	65	100	0.05
**barcode13**	5,809,702	5,809,702	1	65	99.99	0.15
**barcode14**	4,648,520	4,638,958	2	51	100	0.39
**barcode15**	4,653,328	4,638,965	2	51	100	0.69
**barcode16**	6,405,181	6,405,181	1	62	100	0.35
**barcode17**	6,038,335	6,038,335	1	65	100	0.02
**barcode18**	5,715,867	5,715,867	1	64	100	0
**barcode19**	4,792,502	4,792,502	1	61	100	0.84
**barcode20**	5,715,873	5,715,873	1	64	100	0
**barcode21**	6,447,974	6,447,974	1	65	100	0
**barcode22**	6,028,436	6,024,163	2	65	100	0.03
**barcode23**	4,793,440	4,793,440	1	61	100	0.85
**barcode24**	4,662,861	4,638,957	2	51	100	0.54
